# Eigenbased Multi-Antenna Spectrum Sensing: Experimental Validation on a Software-Defined Radio Testbed †

**DOI:** 10.3390/s26051406

**Published:** 2026-02-24

**Authors:** Daniel Gaetano Riviello, Giusi Alfano

**Affiliations:** 1 Department of Electronics and Telecommunications (DET), Politecnico di Torino, 10129 Turin, Italy; 2 CNR-IEIIT, Istituto di Elettronica e di Ingegneria dell’Informazione e delle Telecomunicazioni, Consiglio Nazionale delle Ricerche, 10129 Turin, Italy; 3 Department Electrical and Information Engineering “Maurizio Scarano”, University of Cassino and Southern Lazio, 03043 Cassino, Italy; giuseppa.alfano@unicas.it

**Keywords:** software-defined radio, 6G-CRN, Spectrum Sensing, RLRT, GLRT, URSP

## Abstract

Spectrum Sensing (SS) is expected to play a crucial role in forthcoming 6G Cognitive Radio Networks (CRNs), where unlicensed users will be able to dynamically access the spectrum and perform opportunistic transmissions without generating interference for licensed users. In this work, we investigate multiple-antenna SS techniques by analyzing the performance of several widely used detection schemes—namely, Roy’s Largest Root Test (RLRT), the Generalized Likelihood Ratio Test (GLRT), the Eigenvalue Ratio Detector (ERD), and the Energy Detector (ED)—under varying false-alarm probabilities and signal-to-noise ratios (SNRs). We assume there are a fixed number of sensors at the secondary-user receiver, namely, four. To evaluate the behavior of these detectors in realistic conditions, we developed a software-defined radio (SDR) testbed using Universal Software Radio Peripherals (USRPs), enabling both primary-user signal transmission and secondary-user data acquisition. The experimental results, illustrated through Receiver Operating Characteristic (ROC) and performance curves, are compared with simulation outcomes. The analysis is complemented by a detailed state-of-the-art listing of the available analytical characterizations of the false-alarm probabilities for the considered SS schemes. In particular, the GLRT false-alarm probability, previously unavailable in explicit form for a four-antenna equipped receiver, is computed as well. These results validate the superior detection capability of RLRT over the other schemes tested, confirming its effectiveness not only in theoretical analysis but also in practical SDR-based implementations.

## 1. Introduction

Cognitive Radio Networking (CRN) [1] was conceived as a paradigm through which to counteract the growing shortage of available spectrum resources by promoting the intelligent coexistence of licensed and unlicensed users [2]. Through dynamic sensing and adaptation, CRN allows secondary systems to opportunistically exploit underused frequency bands without causing harmful interference for primary users. Owing to these capabilities, CRN is expected to become a key enabling technology for future 6G communication infrastructures, which will demand unprecedented flexibility, spectrum efficiency, and context awareness.

At the heart of CRN’s functionality lies Spectrum Sensing (SS), a critical process responsible for identifying whether a given frequency band is occupied by a primary transmitter (the busy condition) or currently vacant (in an idle state). The reliability of this sensing process directly impacts spectrum utilization efficiency and interference avoidance. As such, the development of accurate, fast, and robust SS algorithms has remained one of the cornerstone challenges in cognitive radio research over the past two decades [3,4,5]. These efforts have led to a wide range of techniques capable of coping with low signal-to-noise ratios (SNRs), channel variability, and practical hardware constraints.

The present work focuses on a measurement-based evaluation of several well-established SS strategies that operate based on the statistical properties of the received signal covariance matrix [6,7,8,9]. In systems equipped with multiple antennas, such eigenvalue-based approaches exploit spatial diversity to enhance detection reliability. The algorithms considered throughout this work include both statistically grounded procedures, such as the Generalized Likelihood Ratio Test (GLRT) [10] and Roy’s Largest Root Test (RLRT) [11], and more heuristic alternatives, such as the Energy Detector (ED) [12] and the Eigenvalue Ratio Detector (ERD) [6]. While the former rely on rigorous estimation-theoretic principles, the latter are often favored for their implementation simplicity in practical scenarios. In particular, in this work, we extend the content in [13] in two main directions:

- With reference to statistically grounded procedures, we provide a detailed analytical characterization of the false-alarm probabilities for all SS schemes presented both at finite size as well as in the asymptotic regime, and, especially for GLRT, we elaborate on the findings of [14] to provide a more explicit performance evaluation for the finite-dimensional case;
- We add a further heuristic scheme, the above mentioned ERD, to the investigation and, after providing a semi-asymptotic analysis of its performance, carry out a performance evaluation of this SS algorithm both experimentally (i.e., using measurements) as well as via simulations.

The practical implementation of CRN principles has been greatly facilitated by the emergence of software-defined radio (SDR) technology [1,15], which offers a highly flexible and reconfigurable platform for real-time signal acquisition, processing, and control (for a recent account on the topic, see [16]). By shifting signal-processing functionalities from rigid hardware components to programmable software modules [17], SDR devices enable dynamic reconfiguration of the radio front end in response to variations in the surrounding electromagnetic environment—an essential requirement for effective CRN [18]. This inherent adaptability allows radios to modify transmission parameters, waveforms, and protocols on the fly, thereby supporting intelligent spectrum awareness and decision making. Among the available SDR platforms, the Universal Software Radio Peripheral (USRP) has gained particular prominence due to its cost-effectiveness, modular hardware architecture, and seamless compatibility with widely used open-source software frameworks [19,20]. These features make the USRP a popular and reliable choice for the prototyping, implementation, and experimental evaluation of CRN functionalities. The close synergy between CRN concepts and SDR platforms further enables the experimental validation of theoretical spectrum sensing and adaptation strategies, effectively bridging the gap between analytical modeling and real-world performance assessment. This convergence marks a significant step toward the realization of more adaptive, autonomous, and intelligent radio systems capable of continuous self-optimization in dynamic wireless environments.

Extensive experimental research has been reported in the literature to validate SS algorithms using SDR-based setups, particularly focusing on well-known implementations of the ED [12,21,22,23] and cyclostationary feature detectors [24]. Building upon such foundational studies, the present contribution offers a comparative performance analysis of eigenvalue-based detection schemes, including the GLRT, the RLRT, and the ERD, alongside the classical ED, using both simulated and experimentally collected datasets. The experimental framework, designed around a USRP-based receiver, facilitates reproducible real-world testing while capturing the stochastic nature of wireless propagation and hardware-induced imperfections.

From an analytical perspective, the statistical performance of the above detectors has been rigorously studied under various assumptions regarding the number of antennas, observation samples, and channel conditions. Random Matrix Theory has emerged as a powerful instrument for deriving closed-form or asymptotic characterizations of key performance indicators, notably the probabilities of false alarms and detection. These analyses typically fall into two complementary regimes. The first corresponds to the finite-sample setting (see, e.g., [25]), where both the number of antennas and the number of time-domain snapshots are limited, allowing for exact, but often cumbersome, derivations. The second regime considers the large-system limit [26], in which the number of sensors and samples grows simultaneously while a constant ratio is maintained. This asymptotic framework provides insightful scaling laws and compact expressions for detection thresholds, offering valuable guidance for system design and parameter tuning.

Both the finite-sized and asymptotic approaches offer insight into the dependence of sensing performance on the SNR, the number of antennas, and the observation interval length. However, due to their simplicity with respect to the finite sample settings, asymptotic formulations have become an indispensable analytical tool, complementing experimental investigations and serving as the foundation for robust CRN algorithm design. The present study contributes to this ongoing line of research by combining theoretical understanding, numerical simulation, and real-world measurements to provide a comprehensive assessment of eigenvalue-based spectrum sensing within a practical multiple-antenna SDR framework.

This work is structured as follows: Section 2 relates the development of the system model and describes the sensing schemes. It also provides an outline of the corresponding analytical performance computation. The experimental setup and numerical results are reported in Section 4, while Section 5 concludes the work.

### Notation

Throughout the paper, we denote vectors and matrices with boldface lowercase and uppercase letters, respectively. A hat on the variable denotes an estimated quantity (either numerically or measurement-based). We use E[·] to denote the statistical expectation. ||·|| denotes the Euclidean norm of a vector, and ${∥\cdot ∥}_{F}$ denotes the Frobenius norm of a matrix, while we use tr(·) to denote the trace of a square matrix. For matrices and vectors, ${\left(\cdot \right)}^{H}$ indicates the conjugate transpose. Finally, |·| is the determinant of a square matrix.

## 2. System Model

We focus on an SS setting where a receiver equipped with *K* antennas collects *N* time samples from each antenna. We use $y\left(n\right)={\left[y_{1}\left(n\right),\ldots ,y_{K}\left(n\right)\right]}^{T}$ to denote the K×1 received vector at time $n\in \left\{1,\ldots ,N\right\}$, with generic entry $y_{k}\left(n\right)$ denoting the discrete baseband complex sample at the *k*-th receiving antenna.

Under $H_{0}$, namely, in the absence of a primary signal, $y_{k}\left(n\right)$ is a vector of complex Gaussian noise samples with zero mean and variance $\sigma_{v}^{2}$

(1) $${y\left(n\right)|}_{H_{0}}=v\left(n\right)$$

where $v\left(n\right)∼N_{C}\left(0_{K\times 1},\sigma_{v}^{2}I_{K\times K}\right)$. Under $H_{1}$, instead, both the primary signal and noise are present; therefore,

(2) $${y\left(n\right)|}_{H_{1}}=x\left(n\right)+v\left(n\right)=hs\left(n\right)+v\left(n\right)$$

where s(n) is the transmitted signal sample, modeled, without a loss of generality, as Gaussian-distributed with zero mean and variance $\sigma_{s}^{2}$, while h is the K×1 unknown complex channel vector, affected by Rayleigh flat fading. The channel is assumed to be memoryless and constant during the detection period; i.e., block fading is assumed. Under $H_{1}$, we define the signal-to-noise ratio (SNR) at the receiver as

(3) $$\rho ≜\frac{{E\;∥x\left(n\right)∥}^{2}}{{E\;∥v\left(n\right)∥}^{2}}=\frac{\sigma_{s}^{2}}{\sigma_{v}^{2}}\frac{{∥h∥}^{2}}{K}.$$

The detector collects the received samples in the K×N matrix:

(4) $$Y≜\left[y(1)\ldots y(N)\right]=\mathrm{hs}+V$$

where $s≜\left[s(1)\ldots s(N)\right]$ is a 1×N signal vector, and $V≜\left[v(1)\ldots v(N)\right]$ is a K×N noise matrix. The sample covariance matrix R of the received samples is therefore given by

(5) $$R≜\frac{1}{N}YY^{H}.$$

In our next analysis, we will make reference to the spectral decomposition of $R=U\Lambda U^{H}$, with U denoting the random unitary matrix of the eigenvectors and Λ corresponding to the diagonal matrix of the random eigenvalues, whose largest value is denoted as $\lambda_{\max}$ and whose smallest value is denoted as $\lambda_{\min}$, throughout the work.

Crucial to our signal detection task is, in particular, the exploitation of the largest eigenvalue. Indeed, with reference to the scenario where a primary signal is present, and upon defining, for given system parameters, the quantities

(6) $$\tau_{1}=K\rho +1,$$

(7) $$\mu_{s}\left(\tau_{1},K/N\right)≜\tau_{1}\left(1+\frac{K}{N(\tau_{1}-1)}\right)$$

and

(8) $$\xi_{s}\left(\tau_{1},K/N\right)≜\tau_{1}\sqrt{1-\frac{K}{N{\left(\tau_{1}-1\right)}^{2}}}.$$

one can describe a phase transition phenomenon for the largest the eigenvalue of R (see [27]), depending on the relative strength of the primary signal, such that, almost surely (a.s.)

(9) $$\left\{\begin{array}{cc} \lambda_{\max}\overset{a.s.}{\to }\sigma_{v}^{2}{\left(\sqrt{N}+\sqrt{K}\right)}^{2}, & \;\mathrm{if}\;\tau_{1}\leq 1+\sqrt{K/N}\\ \lambda_{\max}\overset{a.s.}{\to }\sigma_{v}^{2}\mu_{s}\left(\tau_{1},K/N\right), & \;\mathrm{if}\;\tau_{1}>1+\sqrt{K/N}. \end{array}\right.$$

## 3. Spectrum-Sensing Schemes and Performance Computation

The test statistic employed by the detector to discriminate between the null hypothesis $H_{0}$ and the presence of the primary signal $H_{1}$ is denoted as *T*; to make a decision, the detector compares it against a pre-defined threshold *t*: if T>t, it chooses $H_{1}$; otherwise, it chooses $H_{0}$. As a consequence, the *probability of a false alarm*

(10) $$P_{\mathrm{fa}}=\mathrm{Pr}\left(T>t|H_{0}\right)$$

and the *probability of detection* is defined as

(11) $$P_{d}=\mathrm{Pr}\left(T>t|H_{1}\right).$$

Hereinafter, we detail the SS schemes exploited in the experimental setups. The first one, the ED, exploits the difference in energy levels corresponding to the two alternative scenarios of the presence vs. absence of a primary signal. In turn, the ERD relies on the increased value of the ratio between the largest and smallest eigenvalue under $H_{1}$ w.r.t. $H_{0}$. The remaining two schemes are based on classical estimation-theoretic criteria and depend on the statistical behavior of the largest eigenvalue of the sample covariance matrix of the collected data.

### 3.1. Energy Detector

The test statistic $T_{\mathrm{ED}}$ computes the average energy of the received signal over the *N* collected samples, normalized by the noise variance $\sigma_{v}^{2}$, namely,

(12) $$T_{\mathrm{ED}}=\frac{1}{KN\sigma_{v}^{2}}\sum_{k=1}^{K}\sum_{n=1}^{N}{\left|y_{k}\left(n\right)\right|}^{2}=\frac{{∥Y∥}_{F}^{2}}{KN\sigma_{v}^{2}}.$$

Note that, since, according to the definition of the Frobenius norm, ${∥Y∥}_{F}^{2}=\mathrm{tr}\left(YY^{H}\right)$, $T_{\mathrm{ED}}$ can be expressed in terms of the spectrum of the sample covariance, as follows:

(13) $$T_{\mathrm{ED}}=\frac{\mathrm{tr}(R)}{K\sigma_{v}^{2}}=\frac{\sum_{i=1}^{K}\lambda_{i}}{K\sigma_{v}^{2}}.$$

For this test, since the law of the normalized trace $T_{n}=\mathrm{tr}\left(R\right)/K$ can be determined, e.g., as in Equation (8) of [14], by solving

(14) $$f_{T_{n}}\left(x\right)=\frac{K^{KN}}{(KN-1)!}x^{KN-1}e^{-Kx},$$

the corresponding $P_{\mathrm{fa}}$ can be written as

(15) $$P_{\mathrm{fa}}=\mathrm{Pr}\left(T_{n}>\sigma_{v}^{2}t|H_{0}\right)=e^{-K\sigma_{v}^{2}t}\sum_{\ell =0}^{KN-1}\frac{{\left(K\sigma_{v}^{2}t\right)}^{\ell }}{\ell !}.$$

Receiver operating characteristic (ROC) curves are computed based on both values of $P_{\mathrm{fa}}$ and $P_{d}$, while the inversion of (15) leads to a proper threshold setting for performance computation. For eigenvalue-based schemes, as anticipated in the Introduction, there will be a need to resort to an asymptotic approach to carry out a performance analysis.

### 3.2. ERD

The idea behind the ERD is that the presence of a primary-user signal has a greater impact on the largest eigenvalue of the covariance of the collected samples than on the corresponding smallest one [6].

As a consequence, the test statistic of this sensing scheme is expressed as

(16) $$T_{\mathrm{ERD}}≜\frac{\lambda_{\max}}{\lambda_{\min}}.$$

Therefore, its probability law coincides with that of the so-called *standard condition number* of the matrix R.

While the statistical distribution of (16) for a dual-antenna receiver (i.e., when K=2) is known thanks to [28], its closed-form expression for arbitrary values of *K* can be found in Theorem 1 of [29].

The corresponding false-alarm probability, for a given threshold t>0, can be written as

(17) $$\begin{array}{c} P_{\mathrm{fa}}=1-\kappa \sum_{\ell =1}^{K}\int_{0}^{+\infty }\det\left(\Theta \left(\lambda ,t\right)\right)d\lambda , \end{array}$$

where

(18) $$\kappa ={\left[\prod_{\ell =1}^{K}\left(N-\ell \right)!\left(K-\ell \right)!\right]}^{-1},$$

and the K×K matrix Θ(λ) has the following entries:

(19) $$\Theta_{i,j}\left(\lambda ,t\right)=\left\{\begin{array}{c} \gamma (N-K+i+j-1,t\lambda )-\gamma (N-K+i+j-1,\lambda ),\;i\neq \ell ;\\ \lambda^{N-K+i+j-2}e^{-\lambda },\;i=\ell . \end{array}\right.$$

In order to assess the analytical performance of the ERD, we resort to an asymptotic approach, where both the number of samples *N* and the number of receiving antennas *K* are assumed to grow large at the same rate; that is, the so-called *aspect ratio* of the random matrix R, e.g., $\frac{K}{N}$, is a number between 0 and 1 (for our purposes, indeed, $0<\frac{K}{N}\ll 1$).

Under this assumption, we can exploit the convergence results for the extreme eigenvalues of R, stating that

(20) $$\lambda_{\max}\overset{a.s.}{\to }b={\left(\sqrt{N}+\sqrt{K}\right)}^{2},$$

(21) $$\lambda_{\min}\overset{a.s.}{\to }a={\left(\sqrt{N}-\sqrt{K}\right)}^{2},$$

while, in distribution, upon defining the scaling parameters

(22) $$\nu =\left(\sqrt{N}+\sqrt{K}\right){\left({\sqrt{N}}^{-1}+{\sqrt{K}}^{-1}\right)}^{1/3},$$

and

(23) $$\mu =\left(\sqrt{K}-\sqrt{N}\right){\left({\sqrt{K}}^{-1}-{\sqrt{N}}^{-1}\right)}^{1/3},$$

both the random variables

$$\ell_{1}=\frac{\lambda_{\max}-b}{\nu }$$

and

$$\ell_{K}=\frac{\lambda_{\min}-a}{\mu }$$

converge to a random variable distributed according to a second order Tracy–Widom distribution (TW2), whose Cumulative Distribution Function (CDF) for the Gaussian Unitary Ensemble (GUE) is explicitly given by

(24) $$F_{TW2}\left(x\right)=\exp\left(-\int_{x}^{\infty }\left(s-x\right)q^{2}\left(s\right)\;ds\right)$$

where q(s) is the unique solution to the Painlevé II differential equation

(25) $$q^{\prime \prime }\left(s\right)=sq\left(s\right)+2q^{3}\left(s\right)$$

satisfying the boundary condition

(26) q(s)∼Ai(s),s→+∞

where Ai(s) denotes the Airy special function, one of the two linearly independent solutions to the following differential equation:

(27) $$z^{\prime \prime }\left(w\right)-wz\left(w\right)=0.$$

As a consequence of the above analysis, the Probability Density Function (PDF) of $T_{\mathrm{ERD}}$ can be written as per Equations (12) and (13) of [6], relying on proper linear transformations of the TW2 law for the involved eigenvalues, namely,

(28) $$f_{\lambda_{\max}}\left(x\right)=\frac{1}{\nu }f_{TW2}\left(\frac{x-b}{\nu }\right),$$

(29) $$f_{\lambda_{\min}}\left(x\right)=-\frac{1}{\mu }f_{TW2}\left(\frac{x-a}{\mu }\right),$$

and approximating, for the sake of simplicity, the behavior of $\lambda_{\max}$ and $\lambda_{\min}$ as if they were independent random variables in order to obtain the PDF and, therefore, the CDF of $T_{\mathrm{ERD}}$, e.g., ${\bar{F}}_{T}$.

Indeed, the PDF of $T_{\mathrm{ERD}}$, e.g., ${\bar{f}}_{T}$, can be obtained via Equation (14) of [6] as:

(30) $${\bar{f}}_{T}\left(t\right)=\left[\int_{0}^{+\infty }x\;f_{\lambda_{\max}}\left(tx\right)\;f_{\lambda_{\min}}\left(x\right)\;dx\right]\cdot I\left(t>1\right),$$

with I(·) being the indicator function of the inner interval. As a consequence of the above computation, threshold setting for ERD is performed upon computing $t_{\mathrm{ERD}}={\bar{F}}_{T}^{-1}\left(1-P_{\mathrm{fa}}\right)$.

### 3.3. RLRT

Roy’s test exploits as test statistics the largest eigenvalue of the sample covariance, normalized by the noise variance. Therefore,

(31) $$T_{\mathrm{RLRT}}≜\frac{\lambda_{\max}}{\sigma_{v}^{2}},$$

$P_{\mathrm{fa}}$ can be immediately computed, for this sensing scheme, as

(32) $$P_{\mathrm{fa}}=1-\mathrm{Pr}\left(\lambda_{\max}<\sigma_{v}^{2}t|H_{0}\right)=1-\kappa \left|\Phi \left(\sigma_{v}^{2}t\right)\right|,$$

with κ as in (18), and where

$${\left[\Phi \left(x\right)\right]}_{i,j}=\left(N+K+i+j-2\right)!\left(1-e^{-x}\sum_{k=0}^{N+K+i+j-2}\frac{x^{k}}{k!}\right).$$

Let us recall that, under $H_{0}$, the CDF of $\lambda_{\max}$ is provided in Corollary 2 of [30], while, under $H_{1}$, it assumes the expression derived in [31].

As discussed, a phase transition threshold exists for the identifiability of a signal with RLRT or GRLT, which can take place only if

(33) $$\tau_{1}>1+\sqrt{K/N}$$

After a phase transition, i.e., in case signals are actually identifiable, the scaled and normalized largest eigenvalue converges in distribution to a standard real Gaussian random variable [32], i.e.,

(34) $$N^{1/2}\frac{\lambda_{\max}-\sigma_{v}^{2}\mu_{s}\left(\tau_{1},K/N\right)}{\sigma_{v}^{2}\xi_{s}\left(\tau_{1},K/N\right)}\to N\left(0,1\right),\;\mathrm{if}\;\tau_{1}>1+\sqrt{K/N},$$

with

(35) $$\mu_{s}\left(\tau_{1},K/N\right)≜\tau_{1}\left(1+\frac{K}{N(\tau_{1}-1)}\right)$$

and

(36) $$\xi_{s}\left(\tau_{1},K/N\right)≜\tau_{1}\sqrt{1-\frac{K}{N{\left(\tau_{1}-1\right)}^{2}}}.$$

In the remainder of this section, for ease of notation, we forgo the dependence on $\tau_{1}$ and K/N in both the expressions of $\xi_{s}$ and $\mu_{s}$.

As a consequence of the convergence results for the largest eigenvalue, it turns out that an approximate expression for the threshold of RLRT, for a given tolerated false-alarm rate of α, is

(37) $$t_{RLRT}\left(\alpha \right)\approx \mu_{s}+F_{TW2}^{-1}\left(1-\alpha \right)\xi_{s}$$

where $F_{TW2}^{-1}$ is the inverse of the TW2 CDF (24). We also remark that, for single-signal detection, the presence of a phase transition phenomenon, for detectability under the alternate hypothesis, can be explicitly formulated in terms of a critical detection threshold, depending on the SNR, as follows:

(38) $$\rho_{Crit}=\frac{1}{\sqrt{KN}}$$

### 3.4. GLRT

In the case of GLRT-based SS, the test statistic, derived by applying the GLR criterion to our detection problem, reads as

(39) $$T_{\mathrm{GLRT}}≜\frac{\lambda_{\max}}{\frac{1}{K}\mathrm{tr}\left(R\right)}.$$

Its analytical characterization turns out to be challenging; however, even though the correlation between $\lambda_{\max}$ and the normalized trace in the denominator of (39) is intractable, as observed in [33], (39) and (13) are statistically independent. Therefore, the computation of an exact expression for both the PDF as well as the CDF for finite values of *K* and *N* has been provided in [14,34] (a thorough analysis of the literature in the asymptotic case can be found, e.g., in [10,26]). With reference to Equation (14) of [14], the false-alarm probability of the GLRT corresponding to a threshold t>0 can be expressed as

(40) $$\begin{array}{c} P_{\mathrm{fa}}=1-\frac{(KN-1)!}{K^{KN-1}}\sum_{i=1}^{K}\sum_{j=N-K}^{\left(N+K\right)i-2i^{2}}i^{KN-j-2}c_{i,j}\left(C\left(t\right)\theta \left(\frac{K}{i}-t\right)+C\left(\frac{K}{i}\right)\theta \left(t-\frac{K}{i}\right)-C\left(1\right)\right), \end{array}$$

where

$$C\left(y\right)={\left(\frac{K}{i}\right)}^{KN-j-2}\sum_{q=0}^{KN-j-1}\frac{{\left(-i/K\right)}^{q}{\left(q+j+1\right)}^{-1}}{(KN-j-2-q)!q!}y^{q+j+1},$$

and the ways of expressing the values of $c_{i,j}$ are reported in the Appendix A. Under $H_{1}$, a finite-sample statistical analysis of the GLRT can be performed by exploiting the results reported in [35], where the random variable $1/T_{\mathrm{GLRT}}$ is characterized. This is the subject of ongoing work. The setting of the threshold for GLRT exploits asymptotic arguments, as done for the RLRT in the previous sub-paragraph, since they are both affected by the phase transition phenomenon, impacting signal detectability. An effective approximation of the $P_{\mathrm{fa}}$ of the GLRT scheme can be found in [26]:

(41) $$\mathrm{Pr}\left[\frac{T_{GLRT}-\mu_{s}}{\xi_{s}}<s\right]\approx F_{TW2}\left(s\right)-\frac{1}{2NK}{\left(\frac{\mu_{s}}{\xi_{s}}\right)}^{2}F_{TW2}^{\prime \prime }\left(s\right),$$

The definitions of $\xi_{s}$ and $\mu_{s}$ are given in the RLRT paragraph. Notice that (41) can be numerically inverted in an efficient way to find the required threshold $t_{GLRT}\left(\alpha \right)$.

### 3.5. Analytical vs. Simulated Statistics

**Figure 1:**
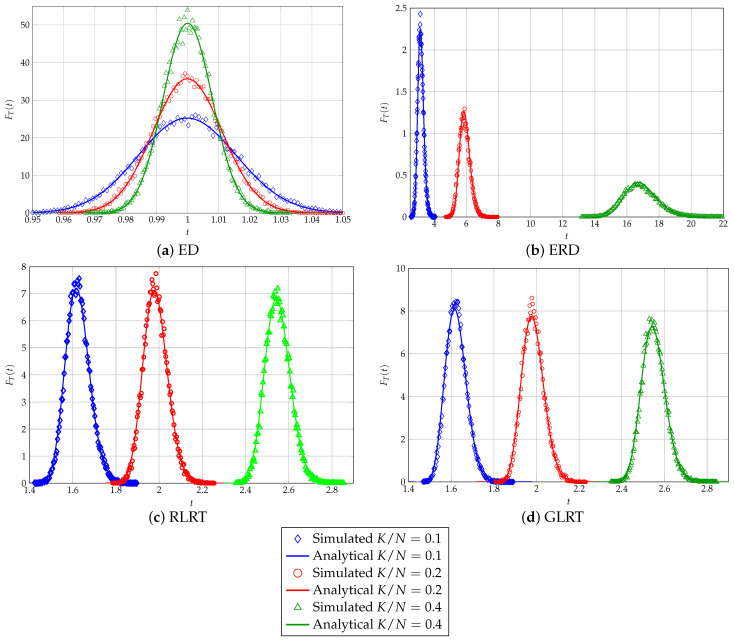
Analytical vs. empirical PDFs of all proposed SS schemes under null hypothesis for different values of K/N.

The degree of matching between analytically computed and numerically simulated statistics of the considered SS schemes has been thoroughly investigated in the literature (see, e.g., [14,26,29]), discussing, in turn, issues regarding computing exact formulae obtained in finite-sized settings or the suitability of asymptotic characterizations for quantifying performance in real scenarios, involving a moderate number of sensors and/or processed samples. For the sake of ensuring the self-consistency of our analysis, in Figure 1, we depict both the analytical PDFs of all the sensing strategies considered (obtained via the formerly described asymptotic procedures), as well as their simulated counterparts, for different values of the ratio K/N, ranging from 0.1 (which is already suitable for resorting to random-matrix-theory-grounded analysis) to 0.4, a *reduced-sample-size* scenario that, while theoretically of interest, does not match usual SS settings, where the number of samples is quite a bit larger than the number of collecting sensors. The sensitivity of the ERD, RLRT, and GLRT to the behavior of the largest eigenvalue of the sample covariance of the channel can be easily remarked, whereas for the ED, only the variance changes, decreasing with increasing values of K/N, while the expected value of the received energy remains constant.

## 4. Experimental Setup and Numerical Results

**Figure 2:**
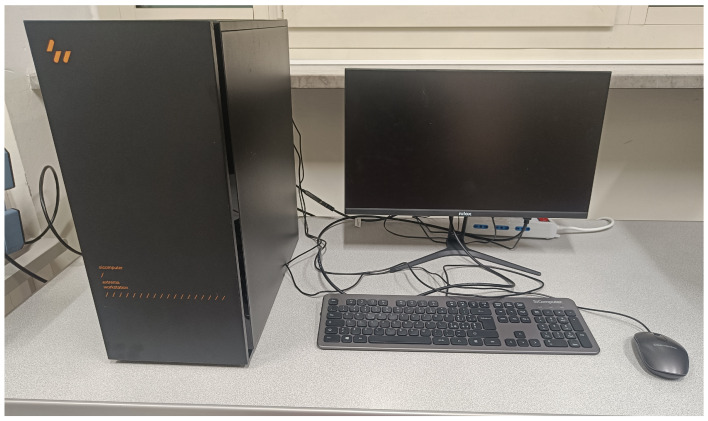
Host computer: SiComputer Extrema Workstations W200.

**Figure 3:**
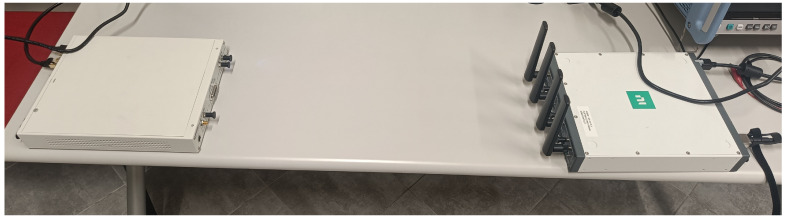
Ettus Research USRP X310, and NI Ettus USRP X410 on the right.

Figure 2 and Figure 3 show the SDR experimental setup, in which the multi-antenna SS algorithms were tested and validated through over-the-air real-time signal transmission and acquisition and subsequent data processing and performance evaluation. The setup is composed of three main components:

- The first component was a host computer, a SiComputer Extrema Workstations W200, equipped with an Intel Core i9-14900K CPU, with 3200 MHz and 24 cores, 64 GB of DDR4 RAM, and a Mellanox ConnectX-5 100 Gbps Ethernet adapter. The SDR software installed was MATLAB R2024b together with the Wireless Testbench support package for NI USRP radios.
- The second was an Ettus Research USRP X310, equipped with a Xilinx Kintex-7 FPGA, with two independent TX/RX channels, capable of streaming up to 200 MSamples/s per channel. The USRP X310 was used as single-antenna transmitter and connected to the workstation through a 10 Gbps Ethernet adapter. Notice that in order to obtain sufficiently low SNR levels suitable for testing the SS schemes at hand, we did not connect an external antenna to the transmitter.
- The third component was an NI Ettus USRP X410, equipped with a Xilinx Zynq Ultrascale+ ZU28DR RF System on Chip (RFSoC), with four independent TX/RX channels, capable of streaming up to 250 MSamples/s per channel. The USRP X410 was used as a multi-antenna receiver and was connected to the workstation through the QSFP28 interface using a 100 Gbps Ethernet adapter.

In order to assess the performance of the RLRT, GRLT, ERD and ED SS schemes, we assumed, as a primary user signal, there was a single carrier QPSK-modulated signal at a carrier frequency of $f_{c}=3.85$ GHz with a baud rate $R_{s}$ of 1 MBd/s. We used four samples per symbol; therefore, the USRP radios were set to a sample rate of $f_{s}=4$ Msamples/s. The narrow band was chosen for the primary signal to ensure there was a flat fading channel. Given the four channels of the USRP X410, K=4, and we performed the experiment for both N=20 and N=40 samples.

Thresholds were computed previously for several values of $P_{\mathrm{fa}}$ and stored offline in a lookup table. While for the ED, the situation is quite straightforward, for RLRT, we used Momar Dieng’s MATLAB package “RMLab” [36], which provides the CDF $F_{TW2}$ of the TW2 distribution. We computed the centering and scaling parameters as in (20) and (22), and then we used the bisection method to retrieve the correct threshold $t_{\mathrm{RLRT}}$ value for any input $P_{\mathrm{fa}}$. For ERD, we first used the same “RMLab” package to compute the scaled and centered PDFs $f_{\lambda_{\max}}\left(tx\right)$ and $f_{\lambda_{\min}}\left(x\right)$ of the TW2 distribution as defined in (28) and (29), respectively, for different values of *t*; secondly, we obtained the PDF of the ERD as in (30) by means of numerical integration for each value of *t*; lastly, we obtained the CDF of ERD by numerically integrating the pdf, and we used the bisection method again to retrieve the correct threshold $t_{\mathrm{ERD}}$ value for any input $P_{\mathrm{fa}}$. Finally, for GLRT, we used the routines in [26], providing the inverted modified distribution of (41).

The SDR experiment can be condensed into three phases:

1. Noise variance estimation under $H_{0}$: In this phase, no transmission occurred, and the USRP X410 acquired samples over its four channels for a total duration of $T_{0}=4$ s. Under the null hypothesis, the Maximum Likelihood estimation of the noise variance $\sigma_{v}^{2}$ can be written as follows:
  (42) $${\hat{\sigma }}_{v}^{2}=\frac{{∥Y|}_{H_{0}}{∥}_{F}^{2}}{KN}.$$
2. Primary user signal transmission and multi-antenna acquisition under $H_{1}$: The USRP X310 was set up to continuously transmit the QPSK-modulated signal, and, simultaneously, the USRP X410 acquired samples under $H_{1}$ again for $T_{0}=4$ s. We can estimate the SNR $\hat{\rho }$ at the receiver using the following approach:
  (43) $$\hat{\rho }=\frac{{∥Y|}_{H_{1}}{∥}_{F}^{2}}{KN\hat{\sigma_{v}^{2}}}-1$$
3. Performance evaluation and comparison with simulated data: In the last phase, we processed the acquired samples into slots of *N* samples, and we computed the RLRT, ERD, GLRT, and ED test statistics. The results were generated through error counting by means of ROC curves for a specific SNR value and by means of a $P_{d}$ vs. SNR curve for a specific $P_{\mathrm{fa}}$ value. In particular, for a fixed $P_{\mathrm{fa}}$, the points of each curve were obtained from the experimental setup by progressively increasing the transmit gain by 0.5 dB at each step and repeating the procedure detailed in these three steps. Experimental results were compared against simulated ones; i.e., we performed a parallel Monte Carlo simulation by generating Gaussian samples with signal variance ${\tilde{\sigma }}_{s}^{2}=\hat{\rho }\;{\hat{\sigma }}_{v}^{2}/{∥h∥}^{2}$, and we applied the same test statistics and metric evaluations.

**Figure 4:**
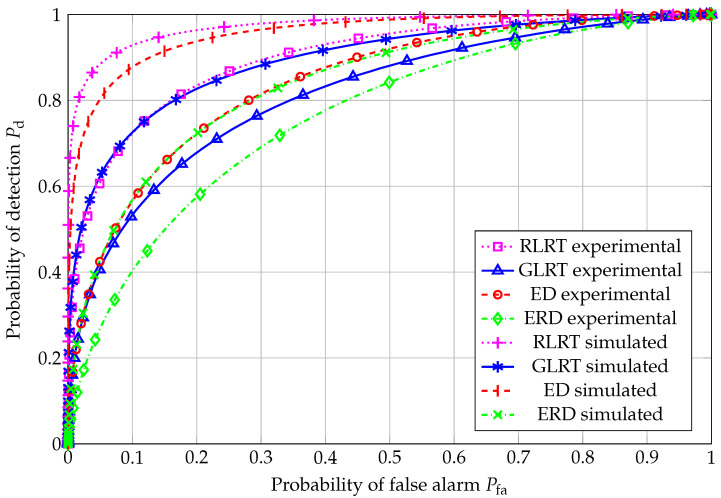
ROC curve: N=20, K=4, and SNR=−4.81 dB.

ROC curves for N=20 are reported in Figure 4, for all the considered SS schemes. Nonidealities in the experimental setup (such as interference during noise estimation, uneven noise floor levels among the four antennas, quantization noise, mulitpath fading, etc.) clearly impact the corresponding performance, which results in poorer performance than the simulated counterparts. As expected, RLRT outperformed all the other schemes, i.e., GLRT, in which there is no prior knowledge of the noise variance; the ED, which cannot make use of any information about the primary signal subspace; and, finally, ERD, which suffers from high noise sensitivity. Another relevant issue affecting the ERD is the underestimation of the denominator with respect to the case of test statistics based on estimation-theoretic criteria, such as the best-performing RLRT and the GLRT as well.

**Figure 5:**
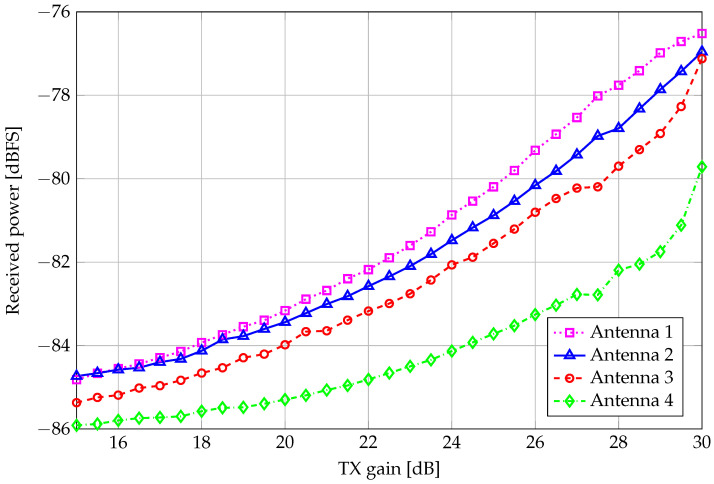
Received signal plus noise power level as a function of the TX gain.

Among all nonidealities, multipath fading should be mitigated by the narrowband signal, while the uneven noise floor levels and received signal powers among the four independent channels play a pivotal role in the performance gap between the experimental and simulated results. A quantitative analysis of the unevenness of the received signal power between USRP antennas is reported in Figure 5, in which the relative full-scale received power levels are plotted against the input transmit radio gain for each USRP channel.

**Figure 6:**
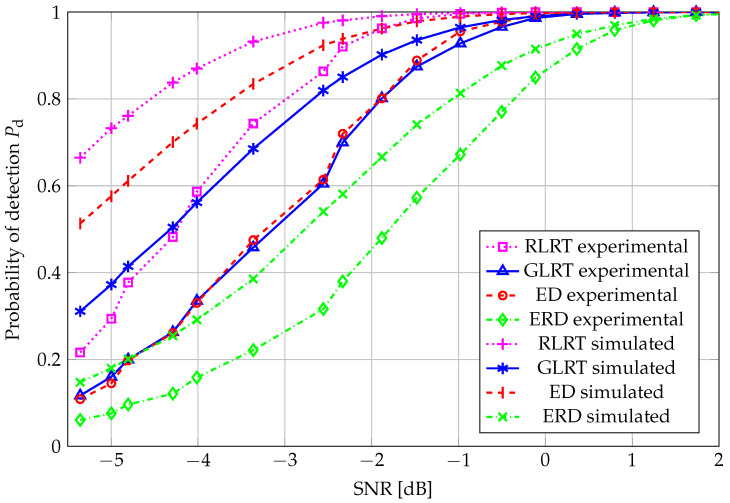
$P_{d}$ vs. SNR: N=20, K=4, and $P_{\mathrm{fa}}=0.01$.

The performance curves representing the behavior of the probability of detection vs. SNR, at a target false-alarm probability of 0.01, are depicted in Figure 6 for N=20. A very similar behavior was observed for all tests, as shown in Figure 4, as the performance ranking among the analyzed algorithms was retained.

**Figure 7:**
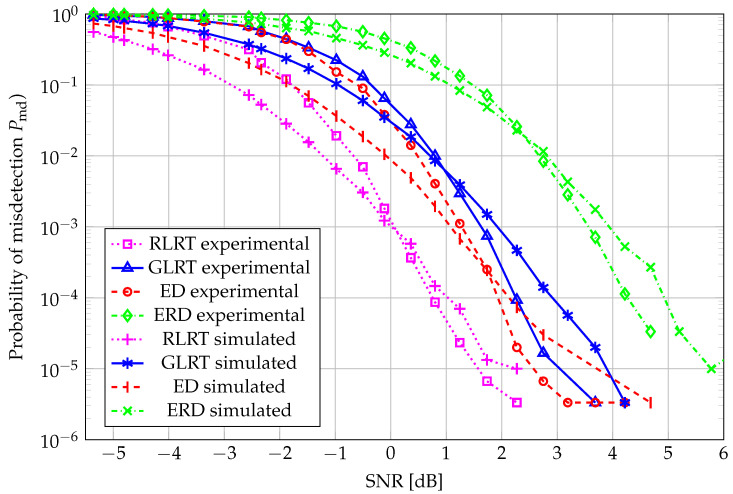
$P_{\mathrm{md}}$ vs. SNR: N=20, K=4, and $P_{\mathrm{fa}}=0.001$.

A complementary perspective, i.e., the probability of misdetection ($P_{\mathrm{md}}=1-P_{d}$) vs. SNR, with $P_{\mathrm{fa}}=0.001$, is reported in Figure 7, wherein, for N=20, the curves do not hold to the already observed behavior over the whole SNR range due to the highly demanding target false alarm, combined with the processing of a small number of samples. However, RLRT still outperforms the remaining schemes, and ERD offers the poorest performance in this case as well.

**Figure 8:**
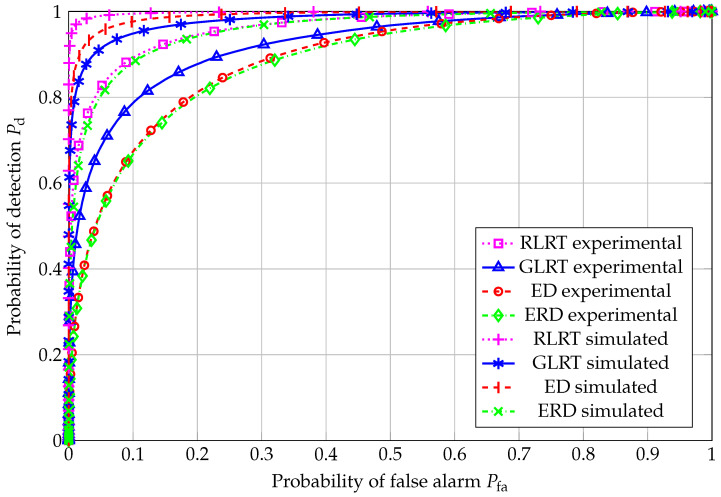
ROC curve: N=40, K=4, and SNR=−4.99 dB.

**Figure 9:**
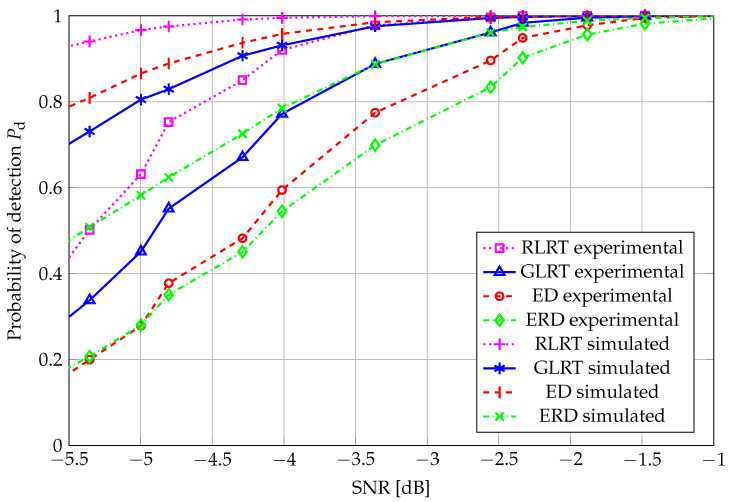
$P_{d}$ vs. SNR: N=40, K=4, and $P_{\mathrm{fa}}=0.01$.

**Figure 10:**
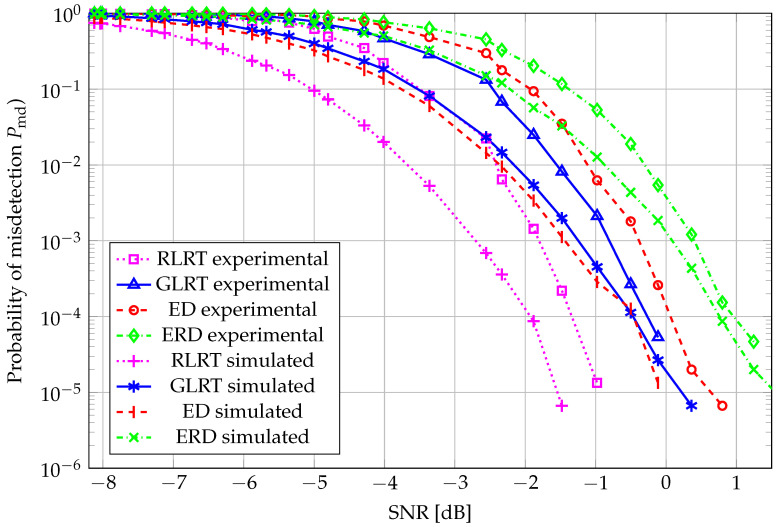
$P_{\mathrm{md}}$ vs. SNR: N=40, K=4, and $P_{\mathrm{fa}}=0.001$.

The analysis is repeated for an increased sample size of N=40; in particular, ROC curves are reported in Figure 8, while the probability of detection vs. SNR is depicted in Figure 9. Performance ranking is unaffected by the increase of the number of collected samples, whereas the absolute performance benefits of the availability of a higher number of data. Remarkably, Figure 10 confirms that the performance ranking is fully retained for N=40.

Overall, the experimental test statistics, with data acquired through SDR platforms, require approximately 0.5–1 more dB to achieve the same performance as the corresponding simulated ones.

**Figure 11:**
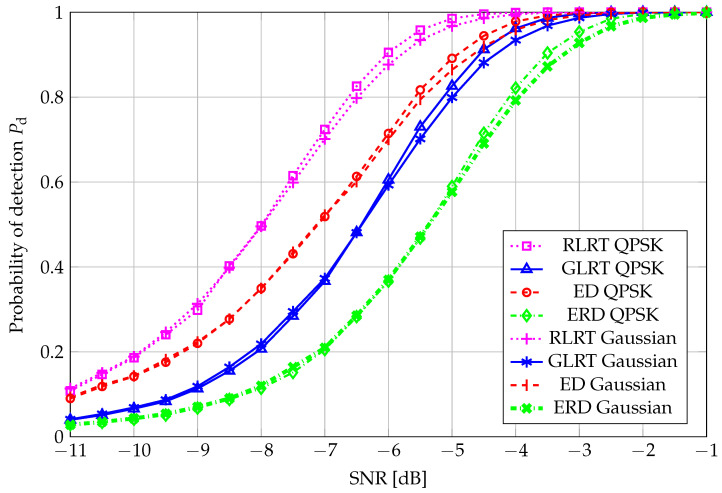
QPSK-modulated vs. Gaussian-distributed primary signal: $P_{d}$ vs. SNR, N=40, K=4, and $P_{\mathrm{fa}}=0.01$.

As a final remark, we evaluated, based on simulated data only, the impact of employing a Gaussian primary signal on the performance of our tests and contrasted this, for each SS scheme, with our main working assumption, i.e., transmitting a QPSK-modulated signal. The curves, reported in Figure 11, confirm that, for the range of values of interest regarding the probability of detection, QPSK transmission is beneficial w.r.t. the use of a Gaussian signal.

## 5. Conclusions

An SDR-based experimental setup was developed to evaluate the performance of multi-antenna SS algorithms under realistic conditions. In this framework, a secondary user terminal equipped with K=4 receiving antennas was employed to collect measurement data. The acquired signals were processed to estimate the false-alarm and detection probabilities associated with four SS schemes, namely, ED, ERD, the GLRT, and RLRT. A thorough review of the mathematical literature was carried out to individuate, whenever available, explicit expressions for the false-alarm performance of each considered scheme, both in the finite-size as well as in an asymptotic regime, where the number of collected samples and that of the receiving sensors both tend toward infinity at the same rate. On top of this, an explicit characterization of the GLRT test statistics in the case of a four-sensor-equipped receiver is provided. The measurement-based performance curves were then compared with the results obtained from numerical simulations. The comparison demonstrates a very close match between experimental and simulated data, confirming the reliability of the proposed setup and the validity of the adopted simulation models. Future research plans include the investigation of finite-size statistical analysis of the presented eigenbased SS schemes under an alternate hypothesis (in the presence of the primary signal); moreover, our presented analysis could be extended to a higher number of sensors, upon a hardware update, and it could include testing on colored noise as well.

## Data Availability

The data that support the findings of this study are available from the first author, D.G.R., upon reasonable request.

## Appendix A

For any positive integer *m*, let us define the polynomial as in Equation (29) of [14]

(A1) $$L_{m}\left(x\right)=2\sum_{k=0}^{2}\frac{{\left(-1\right)}^{k}\left(m-k+2\right)!}{k!(2-k)!}x^{k}-e^{-x}m!\sum_{k=0}^{m}\frac{(m-k+2)!}{k!(m-k)!}x^{k},$$

and recall the expression of the normalizing constant, corresponding to the case we are interested in, namely,

(A2) $$D_{4,N}=\frac{1}{3!(N-1)!(N-2)!(N-3)!(N-4)!}$$

An expression for the PDF of (39), corresponding to K=4, is obtained from Equation (33) of [14] as follows:

(A3) $$\begin{array}{c} f\left(x\right)=D_{4,N}\;x^{N-4}e^{-x}[2\;L_{N-1}\left(x\right)L_{N-2}\left(x\right)L_{N-3}\left(x\right)+L_{N}\left(x\right)L_{N-2}\left(x\right)L_{N-4}\left(x\right)\\ \;\;\;\;\;\;\;\;\;\;\;-L_{N-3}^{2}\left(x\right)L_{N}\left(x\right)-L_{N-1}^{2}\left(x\right)L_{N-4}\left(x\right)-{\left(L_{N-2}\left(x\right)\right)}^{3}]. \end{array}$$

In [14], the suggested roadmap for obtaining the explicit expression of the coefficients of interest requires one to first expand all products of the polynomials appearing in (A4); unless otherwise stated, the products of polynomials of the type

$$L_{a}\left(x\right)L_{b}\left(x\right)L_{c}\left(x\right),$$

with a,b,c∈{N−4,N−3,N−2,N−1,N}, are to be simplified with the help of Equation (34) of [14]:

(A4) $$\sum_{r=0}^{a}p_{r}x^{r}\sum_{r=0}^{b}q_{r}x^{r}\sum_{r=0}^{c}l_{r}x^{r}=\sum_{r=0}^{a+b+c}\sum_{t=\max\{0,r-c\}}^{\min\{r,a+b\}}\sum_{k=\max\{0,t-b\}}^{\min\{t,a\}}p_{k}q_{t-k}l_{r-t}x^{r}$$

After collecting all coefficients for each term of the type $x^{r}e^{-ix}$, i=1,…,4 and applying the identity principle of polynomials to the newly grouped coefficients and to the Equation (6) of [14], it turns out that the required value of the $c_{i,j}$’s in (40), for i=1,…,4 and all corresponding values of *j*, is given by the coefficient of $x^{j-(N-4)}$ in the simplified polynomial according to (A4).

We remark that this procedure works only for K=4. For lower values of *K*, fully explicit expressions of the $c_{i,j}$’s are reported from Equations (23) to (26) of [14], wherein the procedure for obtaining the expressions for K=4 is also outlined, without identifying the degree of the coefficients to be taken into account to finalize the computation.

For K≥5, the $c_{i,j}$’s can be computed via a numerical algorithm, introduced and exploited in [37,38].
